# Impact of autologous whole blood administration upon experimental mouse models of acute *Trypanosoma cruzi* infection

**DOI:** 10.1186/s40409-018-0157-8

**Published:** 2018-08-30

**Authors:** Beatriz Philot Pavão, Kelly Cristina Demarque, Marcos Meuser Batista, Gabriel Melo de Oliveira, Cristiane França da Silva, Francisca Hildemagna Guedes da Silva, Luzia Fátima Gonçalves Caputo, Cynthia Machado Cascabulho, Marcello André Barcinski, Maria de Nazaré Correia Soeiro

**Affiliations:** 1 Laboratório de Biologia Celular, Instituto Oswaldo Cruz, Fundação Oswaldo Cruz, Av. Brasil, 4365, Manguinhos, Rio de Janeiro, RJ Brazil; 2 Laboratório de Patologia, Instituto Oswaldo Cruz, Fundação Oswaldo Cruz, Av. Brasil, 4365, Manguinhos, Rio de Janeiro, RJ Brazil; 3 Laboratório de Inovações em Terapias, Ensino e Bioprodutos, Instituto Oswaldo Cruz, Fundação Oswaldo Cruz, Av. Brasil, 4365, Manguinhos, Rio de Janeiro, RJ Brazil

**Keywords:** Autologous blood, Alternative therapy, Mouse models, *Trypanosoma cruzi*

## Abstract

**Background:**

Autologous whole blood (AWB) administration is described as alternative/complementary medical practice widely employed in medical and veterinary therapy against infections, chronic pathologies and neoplasias. Our aim is to investigate *in vivo* biological effect of AWB using healthy murine models under the course of *Trypanosoma cruzi* acute infection.

**Methods:**

The first set of studies consisted of injecting different volumes of AWB and saline (SAL) into the posterior region of quadriceps muscle of healthy male Swiss mice under distinct therapeutic schemes evaluating: animal behavior, body and organ weight, hemogram, plasmatic biochemical markers for tissue damage and inflammatory cytokine levels and profile. To assess the impact on the experimental *T. cruzi* infection, different schemes (prior and post infection) and periods of AWB administration (from one up to 10 days) were conducted, also employing heterologous whole blood (HWB) and evaluating plasma cytokine profile.

**Results:**

No major adverse events were observed in healthy AWB-treated mice, except gait impairment in animals that received three doses of 20 μL AWB in the same hind limb. AWB and SAL triggered an immediate polymorphonuclear response followed by mononuclear infiltrate. Although SAL triggered an inflammatory response, the kinetics and intensity of the histological profile and humoral mediator levels were different from AWB, the latter occurring earlier and more intensely with concomitant elevation of plasma IL-6. Inflammatory peak response of SAL, mainly composed of mononuclear cells with IL-10, was increased at 24 h. According to the mouse model of acute *T. cruzi* infection, only minor decreases (< 30%) in the parasitemia levels were produced by AWB and HWB given before and after infection, without protecting against mortality. Rises in IFN-gamma, TNF-alpha and IL-6 were detected at 9 dpi in all infected animals as compared to uninfected mice but only Bz displayed a statistically significant diminution (*p =* 0.02) in TNF-alpha levels than infected and untreated mice.

**Conclusions:**

This study revealed that the use of autologous whole blood (AWB) in the acute model employed was unable to reduce the parasitic load of infected mice, providing only a minor decrease in parasitemia levels (up to 30%) but without protecting against animal mortality. Further *in vivo* studies will be necessary to elucidate the effective impact of this procedure.

## Background

Alternative or complementary medical practices (ACMPs) encompass the medical and veterinary use of therapies that are not routinely offered by and/or accepted by the traditional care systems, but that has attracted the attention of thousands of people in different countries [1, 2]. The ACMPs include different interventions such as electrotherapy [3–5], Ayurveda [6], biofeedback [7], hypnosis [8], “energy healing therapy”/Reiki [9], special diets (such as vegetarian and macrobiotic) [10], Yoga [11], autohemotherapy [12], and homeopathy [13] among others [14, 15]. Due to their low costs, the use of ACMPs, once approved by rigorous pre-clinical and clinical studies, may represent an important complementary approach for the treatment of e.g., the so-called, orphans and/or neglected diseases such as Chagas disease [16, 17], Leishmaniasis [18] and Human African trypanosomiasis [19] that affect large populations living in very poor areas of the world. In this context, the use of autologous whole blood (AWB) has been described as an alternative and/or complementary medical [20] and veterinary [21, 22] intervention against several pathologies such as those caused by an infectious agent [23], due to its autoimmune origin [24], as well as chronic and degenerative inflammation [12, 25] or malignancy [13, 26–28].

Autohemotherapy – also known as autologous whole blood (AWB) intervention, serum therapy, immunotherapy or autohemotransfusion – was proposed by Ravaut, about one century ago, as a therapeutic approach toward different human pathological conditions [29]. AWB has been used under different modalities employing distinct administration routes (intravenous (iv), intraarticular (iar), intramuscular (im), subcutaneous (sc), intra-arterial (ia), and others), and volume managements with or without previous ozone incubation [27, 30, 31]. The most widespread form is the withdrawal of venous blood followed by immediate intramuscular administration. However, there is an important gap as to the mechanisms of action. No clear consensus exists on the AWB mechanistic event despite the proposal of several mechanisms including: (i) improvement of the micro-circulatory system, such as (ii) increase in oxygen concentration in ischemic tissues, (iii) enhancement of the glycolytic pathway of erythrocytes, (iv) stimulation by “physiological mode” of the host immune response and (v) modulation of oxidative balance, and others, depending on the procedure (e.g., with or without ozone), the volume administered (“minor” - 5-20 mL or “major” - 200-400 mL), route of administration (iv, im, sc, etc.) besides the nature of the pathology [25, 27–29, 32, 33].

Therefore, despite the use of this practice by thousands of individuals on different continents, with some reports of improvement of clinical aspects in patients [20–22, 34], there is still a considerable lack of clinical and preclinical studies related to AWB that could elucidate action mechanisms and that could demonstrate its effectiveness and safety profiles [35–38].

Murine models have been employed to explore several pathological aspects including those related to parasitic infection [39]. Furthermore, these models have been used in other studies to evaluate the impact of this whole blood therapy [32, 38, 40]. In this context, our aim was to evaluate, through pre-clinical assays using mouse experimental models, the biological effect and potential side effects of the autologous whole blood administration in healthy animals, exploring some clinical and histopathological *in vivo* aspects. Also investigated was the potential impact of AWB upon an infection condition, namely that caused by the intracellular parasite *Trypanosoma cruzi*, the etiological agent of Chagas disease, using a mouse model of acute parasitic infection.

## Methods

### Animal models and ethics

Male Swiss mice obtained from the Fundação Oswaldo Cruz (FIOCRUZ) animal facilities (Rio de Janeiro, Brazil) were housed at a maximum of six per cage and kept in a conventional room at 20–24 °C under a 12/12 h light/dark cycle. The animals were allowed to acclimate for 7 days before starting the experiments and were provided with sterilized water and food ad libitum. The animals were subjected to a randomization procedure for animal (18–20 g) distribution in the different groups and all studies were carried out using in parallel untreated (animals only bled or neither bled nor injected) and saline-treated mice (10 or 20 μL of NaCl 0.85%) as controls. The number of animals per group was always at least 3 (minimum of 3 and maximum of 10) in order to provide replicable data [41]. The experimental protocol was approved by the Ethics Commission on Animal Use of the Oswaldo Cruz Institute (CEUA/IOC number CEUA L-032/2016), following Brazilian law and the recommendations of the National Commission for Ethics in Research (CONCEA), in accordance with the International Guiding Principles for Biomedical Research Involving Animals.

### Parasites and infection of mice

For all the assays, bloodstream trypomastigotes (BT) of the Y strain were used throughout and were harvested by heart puncture from *T. cruzi*-infected Swiss mice on the parasitemia peak day, as previously described [42]. Swiss Webster male mice (38.6 ± 2.6 g) were infected by i.p. injection of 10^4^ BT (Y strain). Age-matched non-infected mice were maintained under identical conditions.

### Treatment schemes

**Fig. 1:**
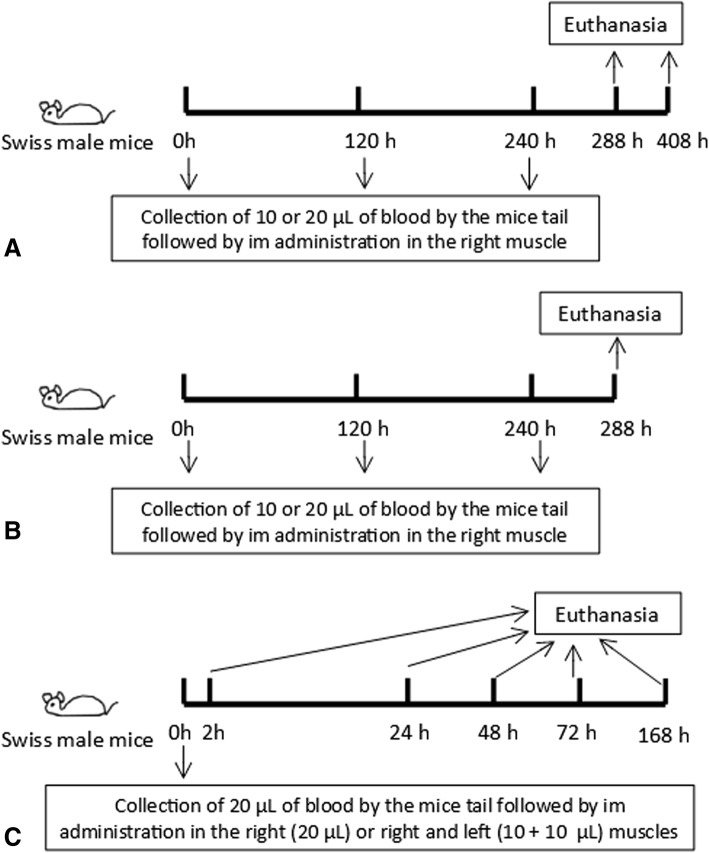
Intramuscular treatment approach according to the different schemes. (**a**) and (**b**) (Scheme 1): three AWB administrations at five-day intervals and (**c**) (Scheme 2): single AWB administration. Control groups: administration of SAL, bled and untreated mice, and groups of non-bled and untreated mice

The experiments carried out using healthy mice were performed by injecting autologous venous blood (previously collected from the same animal tail) into the quadriceps muscle of the back of thigh hind limbs (10 and 20 μL into the right muscle or 10 μL of each into the left and right). Two different sets of protocols were conducted (Fig. 1): (i) three single administrations of saline and AWB samples at 5-day intervals between each im administration, then evaluated at 48 and 168 h after the last administration (Fig. 1a and b); and (ii) single treatment followed by the analysis from 2 up to 168 h after injection (Fig. 1c). Primary outcomes (body weight, animal behavior, clinical side effects and survival rates) were analyzed throughout the assays. At the endpoint (2–168 h post injection), healthy mice were euthanized and the following procedures performed (secondary outcomes): (i) blood collection for analysis of complete blood count (CBC) and analysis of biochemical markers of tissue lesions and inflammatory mediators profile, and (ii) whole thighs for histopathological analysis of different parameters such as inflammatory infiltrate and tissue lesion grade. In the first analysis (first and second assay) all mice were injected using an insulin syringe (13 × 0.45 mm needle (26G), while in the third experiment, using healthy animals, *T. cruzi*-infected mice were treated using a BD Ultra-Fine with 6 × 0.25 mm needle (31G).

**Fig. 2:**
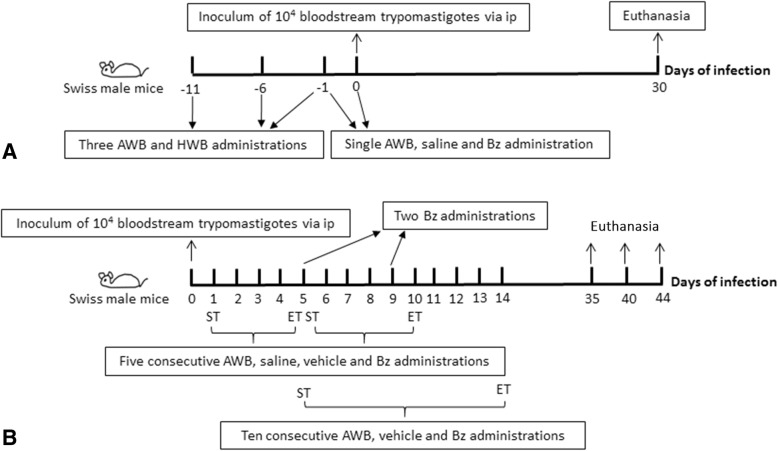
Schemes employed before (**a**) and after (**b**) *T. cruzi* acute infection (Y strain) of mice. The therapy was given intramuscularly (autologous whole blood – AWB and heterologous whole blood – HWB) and orally (benznidazole – Bz) using from one up to 10 daily administrations. Control groups: saline, Bz or vehicle, infected-and-untreated and uninfected-and-untreated. ST = Start of therapy, ET = End of therapy

For the analysis of parasitic infection, mice were inoculated intramuscularly (as described above) with 20 μL AWB or heterologous whole blood (HWB), previously collected from the animals’ tail. The HWB treatment consisted of collection (donor - animal 1) and blood administration (recipient - animal 2) (and vice versa), always using the same donors and recipients for the blood exchanges with animals previously tagged with picric acid (*n* = 1 marking on the head, *n* = 2 on the back, *n* = 3 on the tail, *n* = 4 on the right anterior limb, *n* = 5 on the left anterior limb and *n* = 6 on the right posterior limb). Control groups consisted of: (i) animals injected with 20 μL of saline (SAL – NaCl 0.85%), (ii) animals treated with benznidazole (N-benzyl-2-nitroimidazole acetamide - Bz, at the optimal dose - 100 mg/kg), (iii) animals that were only infected and untreated, (iv) animals that were only infected and treated with vehicle (Tween 80, p.o) and (v) animals neither infected nor subjected to any type of intervention. The parasitemia, mortality rates and body weight were analyzed throughout the assays and at the endpoint (30 days after treatment) with a different set of protocols (Fig. 2): (i) Prior animal infection (Fig. 2a) by single administration at 2 and 24 h or by multiple administrations (three times at 5-day intervals) 24 h before the parasite inoculation, and (ii) After animal infection using multiple administrations (up to 10 consecutive days) started at 1 or 5 days post infection (dpi) (Fig. 2b). In all assays, only mice with positive parasitemia were used in the infected groups. As reference drug for Chagas disease, Bz was used and purchased from Laboratório Farmacêutico do Estado de Pernambuco (LAFEPE), Brazil. The stock solution was prepared in sterile distilled water with 3% Tween 80 (Sigma-Aldrich); before use; it was diluted in sterile distilled water for p.o. administration [41].

### Biochemical and complete blood count analysis (CBC)

At each endpoint, biochemical and CBC analyses were conducted using blood samples from uninfected mice collected through heart puncture. All biochemical analyses were performed at animal facilities of the Oswaldo Cruz Foundation (Rio de Janeiro, Brazil, ICTB/Fiocruz platform) including determination of plasma tissue markers such as urea (BUN), alanine aminotransferase (ALT), aspartate aminotransferase (AST) and creatine kinase (CK) using Vitros 250 (Ortho Clinical-Johnson & Johnson), as reported previously [43]. The results are expressed as enzyme concentration (U/L) and mg/dL (for urea analysis). In all assays, non-treated and treated groups were compared using analysis of variance (ANOVA) and results considered statistically significant at *p* ≤ 0.05. In order to differentiate the leukocyte populations, blood smears collected through the animal tail vein were prepared and inspected individually before and at the endpoint. The samples were stained with Giemsa and the quantification performed under light microscopy to determine the percentages (mean and SD) of lymphocytes, neutrophils, monocytes, eosinophils and basophils. For this analysis, one assay was performed for each treatment protocol (*n* = 2–5 each group).

### Behavioral analysis

The behavior of healthy animals (in Scheme 1 – assays 1 and 2) was tested in a climatized room. To characterize the spontaneous activity of the animals, we used the video-tracking tool Noldus EthoVision XT6 (Noldus Information Technology, Leesburg, Netherlands). The arena was defined as 12 rectangles, divided into lateral and central areas. In the total arena, the rectangles were calibrated with equal areas to ensure the consistency of the parameters with which the apparatus detected transitional mouse movements. This analysis measured the following parameters at different times: (i) locomotor activity, i.e., covered distance (m) and average velocity (cm/s); and (ii) exploratory activity, the frequency of travel to the central region (number of events) every 5 min and the time spent in this region (seconds). The different groups were compared using the Student’s t-test with the results considered statistically significant at *p* ≤ 0.05 [44]. The tests were performed twice for control groups (neither bled nor injected), AWB 20 μL and saline 20 μL analysis (*n* = 10 each group).

### Histological analysis

At each endpoint, besides the gross pathology analysis, the samples from healthy mice were removed and fixed with 10% formaldehyde in PBS solution, decalcified in 10% ethylenediaminetetraacetic acid (EDTA) and processed routinely for histologic evaluation (paraffin embedding technique). Sections (5 μm) stained by routine hematoxylin-eosin (HE) were analyzed by light microscopy. The extent of inflammatory infiltrates (more than 10 infiltrating cells) was determined in at least 5–10 fields from images captured by light microscopy (total magnification, 100×) Axio Observer.A1 (Carl Zeiss). For each slide, at least three sections from each mouse were evaluated. The results from analysis of variance (ANOVA) were considered statistically significant at *p* ≤ 0.05. For those animals that exhibited inflammatory infiltrates with predominant profile of polymorphonuclear cells, an additional staining was performed using the Sirius red method that allows the identification of eosinophils (counted in at least 100 inflammatory cells) [45]. The tissue injury grade was also employed to characterize the extension of the inflammatory infiltration (focal versus diffuse) using the following classification: 0 = no change, 1 = mild localized inflammatory infiltrate, 2 = mild multifocal inflammatory infiltrate, 3 = moderate localized inflammatory infiltrate, 4 = moderate multifocal inflammatory infiltrate, 5 = severe and diffuse inflammatory infiltrate. For this analysis, one assay was performed for each treatment protocol (*n* > 3 per group, in each protocol).

### Animal body and organ weight and survival rates

Body weight variation and mortality rates in both healthy and *T. cruzi-*infected groups were checked individually weekly and daily, respectively. In the assays performed on healthy animals, at the each endpoint, the heart, spleen, liver and kidneys were collected and their respective weights measured [44]. In all assays, the different groups were compared using analysis of variance (ANOVA) and the results considered statistically significant when *p* ≤ 0.05. For this analysis, two assays were performed for each treatment protocol (*n* = 10 regarding three-administration and *n* = 3 for one-administration protocol).

### Cytokine analysis

The profile analysis of cytokines from healthy (one assay with *n* = 2–3 each group) and *T. cruzi*-infected (one assay with *n* = 2–5 each group) mice was conducted by flow cytometry using plasma samples obtained from blood collected through heart puncture. A Cytometric Bead Array kit (BD Biosciences, San Jose, CA) was used for interleukin (IL)-17A, IL-10, interferon (IFN)-g, tumor necrosis factor (TNF, IL-6, IL-4 and IL-2 quantification, according to the manufacturer’s instructions. The samples were acquired in a FACSCalibur flow cytometer (BD Biosciences) and data analysis performed using the software FCAP (BD). In all assays, the different groups were compared using analysis of variance (ANOVA) or Kruskal-Wallis test and the results considered statistically significant when *p* ≤ 0.05.

### Parasitemia, mortality rates and ponderal-curve analysis

The level of *T. cruzi* parasitemia was measured by the Pizzi-Brener method. Mice were individually checked by direct microscopic counting of parasites in 5 μl of blood [46]. Animal weight was determined weekly in each group [47]. In all assays, the different groups were compared using analysis of variance (ANOVA) or Kruskal-Wallis test, and the results considered statistically significant at *p* ≤ 0.05. The tests were performed with one assay for each treatment protocol (except for the group treated with three administrations of AWB before infection, with two assay repetitions), with *n* = 5–6 each group.

## Results

### Analysis of AWB administration in healthy animals

The first step evaluated the impact of AWB upon clinical aspects of healthy animals using different therapeutic schemes. In scheme one, the animals received three injections of 10 and 20 μL of AWB and SAL (total volumes that not exceeded 1 mL/kg). Neither AWB nor SAL induced differences in the animal weight gain or in the size or weight of the heart, spleen, liver or kidneys (data not shown). As to animal behavior, no major alteration was noted in either exploratory or motor activities, except for gait impairment in 20% of those animals that received 20 μL of AWB in the same quadriceps muscle (data not shown). At 48 h after the third autologous blood administration, the cell blood counting (CBC) analysis showed a reduced level of leucocytes (WBC) when compared to control group (that did not receive any kind of intervention), being more evident in those animals that received 20 μL of AWB, although still within the range of the reference values. The statistical analysis showed that the values of erythrocytes (RBC) were significantly (*p* ≤ 0.05) lower in all groups that had received any type of intervention (including those only bled). The number of platelets presented a statistically significant decrease in the group receiving 20 μL of blood (Scheme 1) at 48 h after the last AWB administration. For the other CBC parameters, no major differences were observed (data not shown).

Following the analysis of plasma biochemical markers to assess potential tissue lesions in healthy mice, only minor and inconsistent alterations were noted, including a reduction of ALT levels after 48 h in the group that received 10 μL of blood (data not shown). A small increase in the urea levels was observed in mice only bled, and in those injected with 10 μL of blood and with 20 μL of saline. The findings observed 168 h after the last injection demonstrated no major differences in the biochemical analysis between the studied groups (data not shown).

**Fig. 3:**
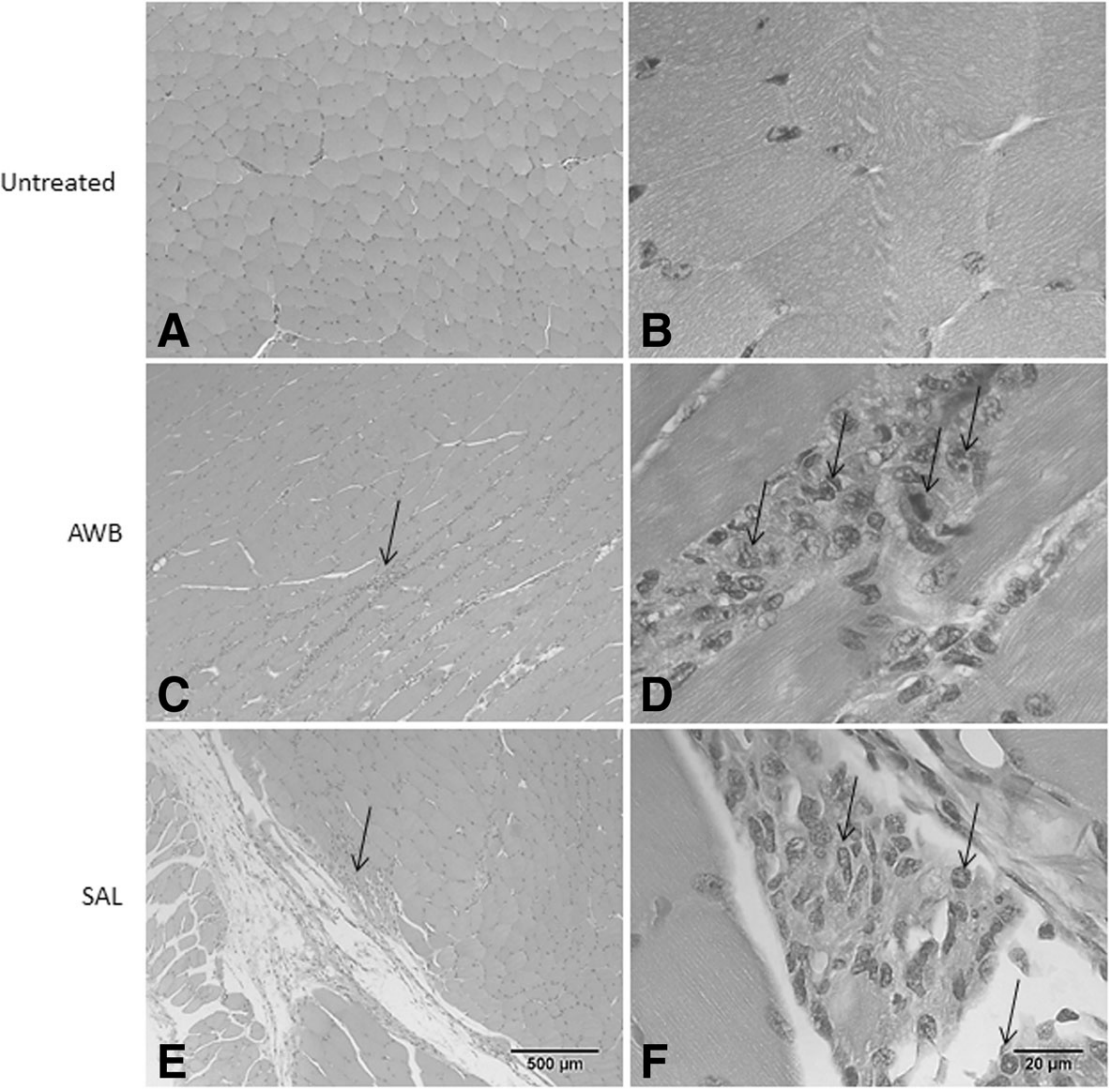
Histopathological analysis of the inflammatory infiltrate profile in uninfected mice. The mice were submitted to three administrations of 20 μL of AWB (**c** and **d**) and of SAL (**e** and **f**) at five-day intervals (Scheme 1). The evaluation by light microscopy was performed by hematoxylin-eosin staining from the posterior region of the quadriceps muscle collected from mice 48 h after AWB and SAL injection (**c**-**f**) compared to samples obtained from untreated (**a**, **b**) animals. Original increments × 100 (**a**, **c** and **e**) and × 1000 (**b**, **d** and **f**). Arrows: inflammatory infiltrate

**Fig. 4:**
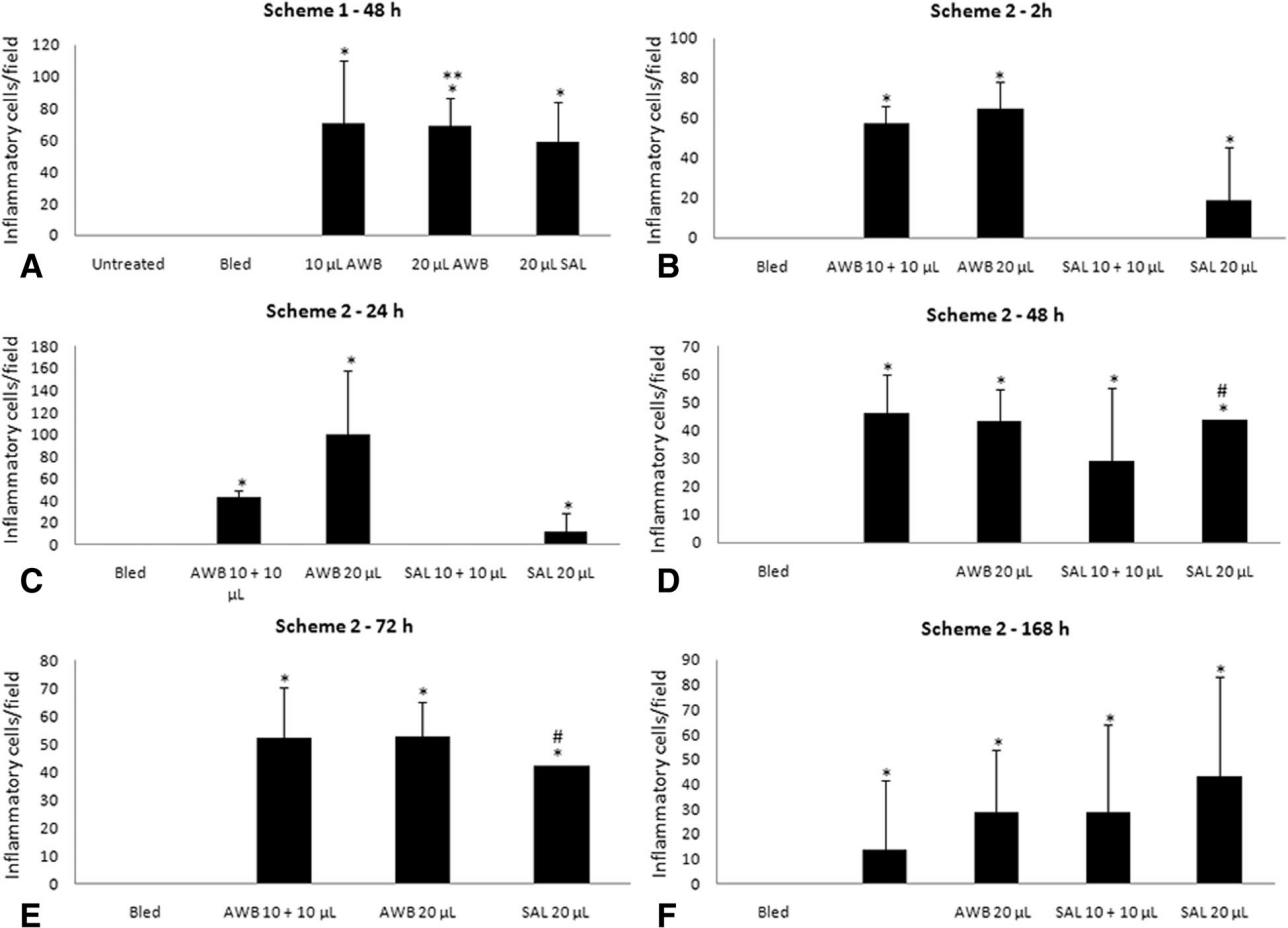
Analysis of the inflammatory presence in thighs of uninfected mice. Evaluation of the number of inflammatory cells (mean and SD) after administration of AWB and SAL at 48 h (**a**) (three administration under a five-day interval - Scheme 1) and at 2 h (**b**), 24 h (**c**), 48 h (**d**), 72 h (**e**) and 168 h (**f**) (unique administration - Scheme 2) post treatment. ANOVA (*p* ≤ 0.05) = *untreated/bled; ** AWB and SAL; # = individual analysis

**Fig. 5:**
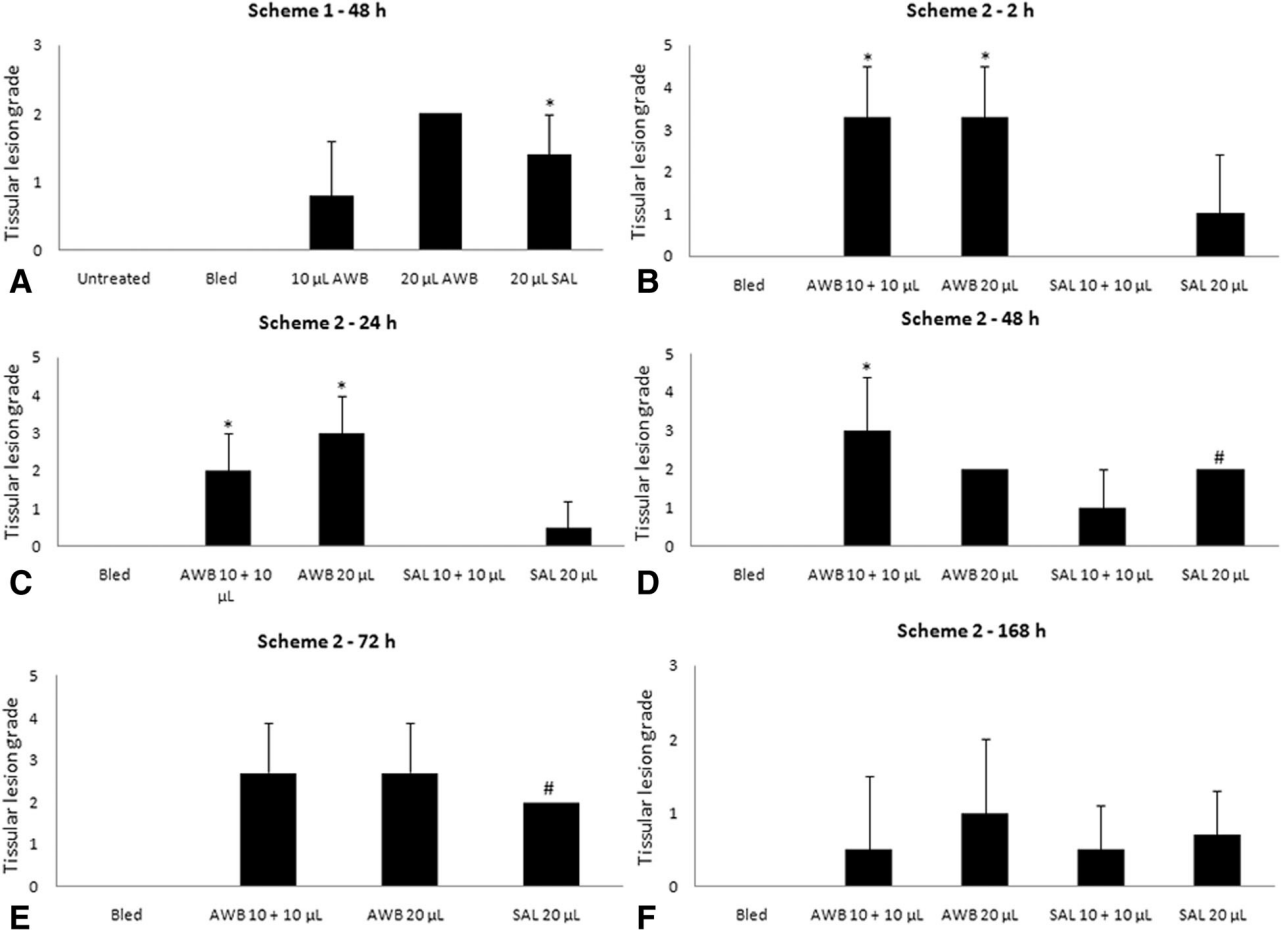
Analysis of tissue lesion in thighs of uninfected mice. Evaluation of degree of tissue lesion (mean and SD) after administration of AWB and SAL at 48 h (**a**) (three administration under a five-day interval - Scheme 1) and at 2 h (**b**), 24 h (**c**), 48 h (**d**), 72 h (**e**) and 168 h (**f**) (unique administration - Scheme 2) post treatment. The tissue injury grade was assessed using the following classification: 0 = no change, 1 = mild localized inflammatory infiltrate, 2 = mild multifocal inflammatory infiltrate, 3 = moderate localized inflammatory infiltrate, 4 = moderate multifocal inflammatory infiltrate, 5 = severe diffuse inflammatory infiltrate. ANOVA (*p* ≤ 0.05) = *untreated/bled; # = individual analysis

Histological analysis of the muscle samples from healthy animals showed an inflammatory infiltrate at 48 h after injection of blood and saline, being much higher in those submitted to AWB administration (Figs. 3, 4a, and 5a). The healthy animals injected with autologous blood (Fig. 3c, d) showed a statistically higher (*p* ≤ 0.05) degree of inflammation and greater number of cells per field than the SAL (Fig. 3e, f) group. At 48 h after injection, except for one animal from the AWB (20 μL), all animals subjected to AWB and SAL intervention showed a high predominance of mononuclear cells in the inflammatory infiltrate (data not shown). At 168 h after the last injection, no inflammatory or lesion signs were detected in muscle samples by histopathology in both SAL and AWB groups and through the biochemical measurements of plasma CK levels (data not shown). Next, assays were conducted using the larger volume (20 μL) and as negative controls, the animals bled but not injected since no major difference were found among the other control groups (Fig. 1). Thus, the following assays using AWB or SAL confirmed previous analysis. At 48 h after the last injection, no major differences were found in the different parameters including body and organ weights (data not shown) or in the biochemical blood analysis (data not shown). As to the exploratory and motor activities, although no relevant differences were found among the studied groups, some animals (40%) that received AWB presented gait impairment in the paw where the administration was performed (data not shown). In order to ascertain whether only one administration of AWB could trigger a tissue inflammatory profile similar to that from repetitive administrations, another set of studies (Scheme 2) was performed on healthy mice injected once with 20 μL of blood and saline, also distributing this volume into the two quadriceps muscles (10 μL of volume in right and left muscles each; Fig. 1).

**Fig. 6:**
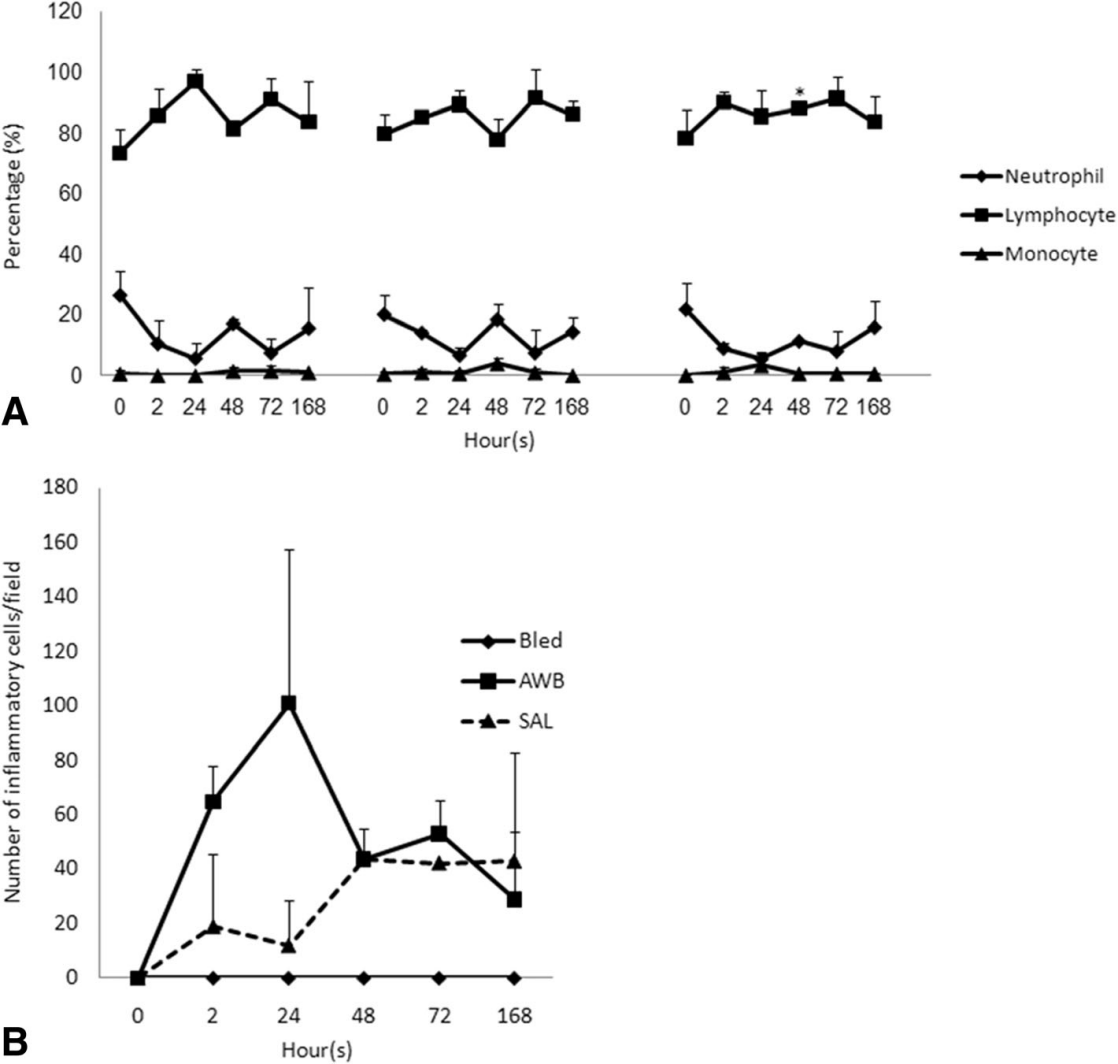
Leukogram and inflammatory tissue analysis in uninfected mice. Mean and SD of (**a**) leukogram by blood smear stained with Giemsa and (**b**) tissue inflammation of mice thighs submitted or not to AWB and SAL injection. The samples were collected at endpoints of 2, 24, 48, 72 and 168 h after treatment (single administration - Scheme 2). * ANOVA = *p* ≤ 0.05 (*n* = 2–3) between bleeding / treated animals

Up to 168 h after the last injection, no group exhibited major differences related to the different parameters evaluated including body and organ weights (data not shown) as well as biochemical blood analysis, except for a decrease in ALT and AST levels after 48 h in those injected with 20 μL of blood (data not shown). Hemogram analysis did not demonstrate major alterations except that all mice having received any type of injection presented higher levels of platelets after 72 h as compared to the control group (data not shown). At each time-point a blood smear analysis using Giemsa stained samples was conducted, whose major difference was the increase of monocyte levels (1–4%) when mice were submitted to AWB injection (Fig. 6a).

**Fig. 7:**
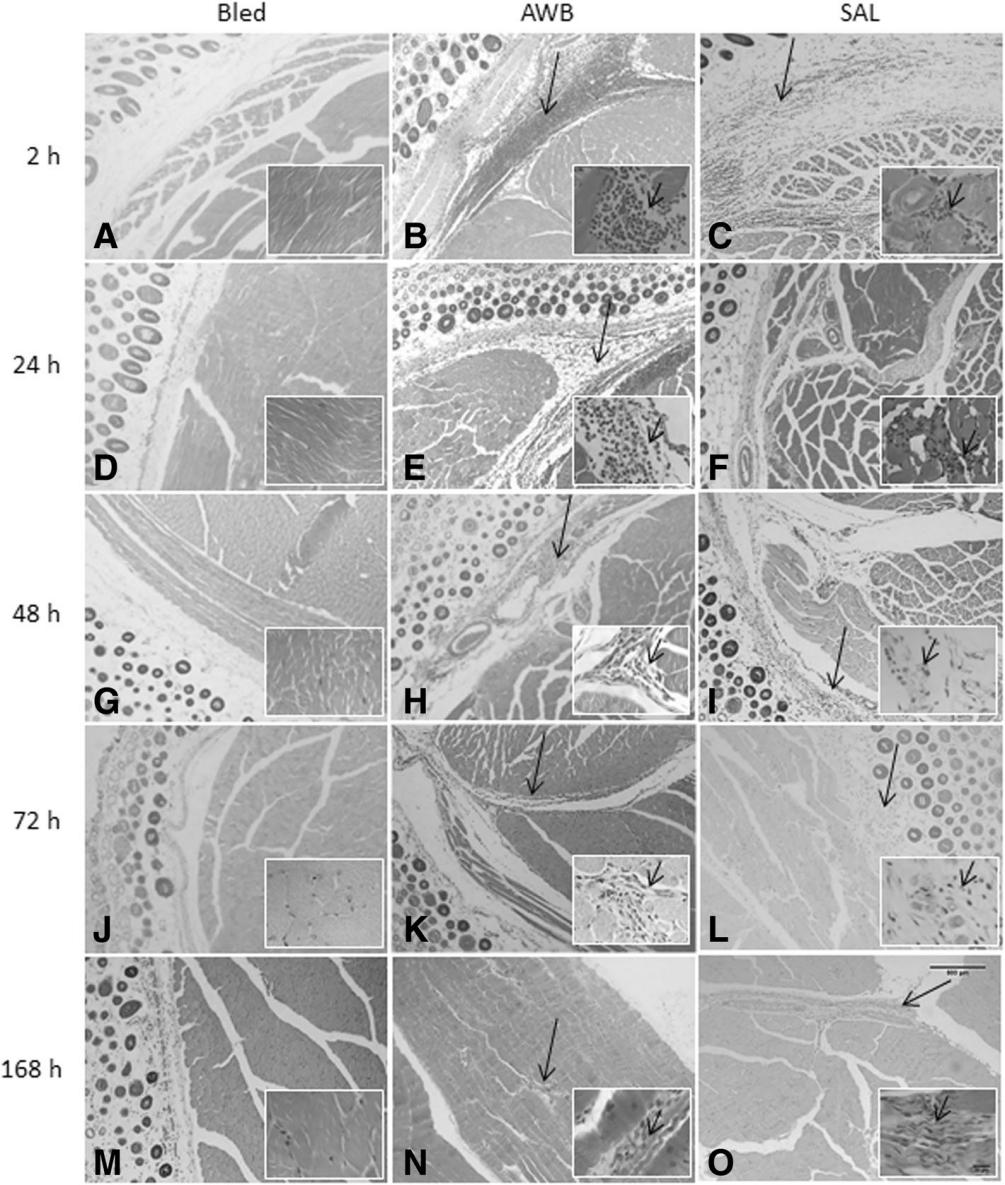
Histopathological analysis of the presence of inflammatory infiltrate in uninfected mice. The evaluation of inflammatory infiltrate was performed by light microscopy (**a**-**o**) using hematoxylin-eosin staining of quadriceps muscles from the back of the hind thigh collected from mice at 2, 24, 48, 72, 168 h after a single injection (Scheme 2) of 20 μL of AWB (**b**, **e**, **h**, **k** and **n**) and SAL (**c**, **f**, **i**, **l** and **o**). Original magnifications × 100 and × 1000 (inset) for all panels

**Fig. 8:**
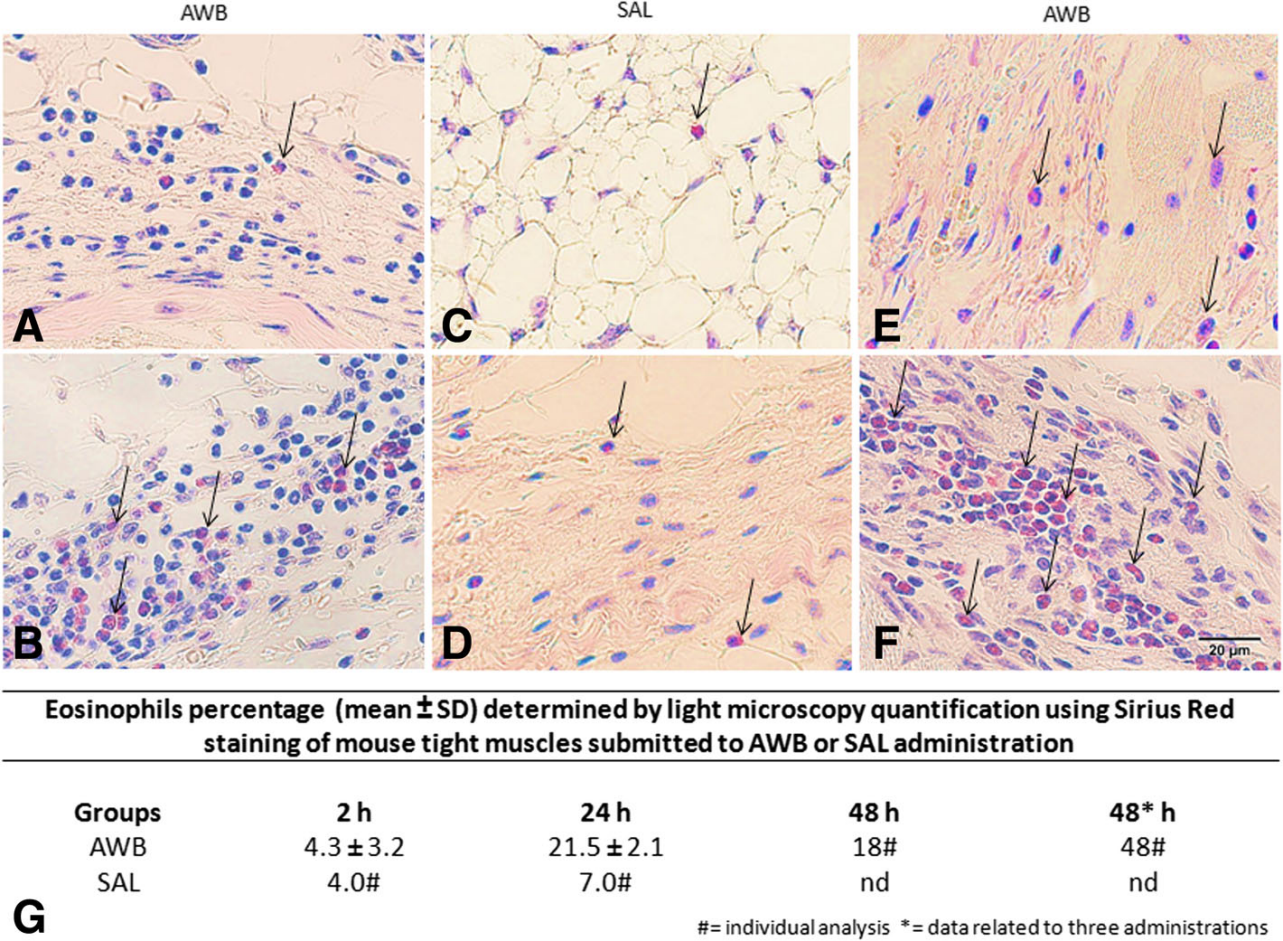
Histopathological analysis of the presence of eosinophils in uninfected mice. The evaluation was performed by using Sirius Red staining of quadriceps muscles from the back of the hind thigh collected from mice submitted to AWB and SAL therapy. (**a**-**f**) Light microscopy analysis and (**g**) determination of the percentage of eosinophils (mean ± SD) stained by the pink labeling (arrows, **a**-**f**) of tissue samples collected from mice submitted to one (**a**-**e**) or three (**f**) cycles of AWB (**a**, **b**, **f** and **g**) and SAL (**c**, **d** and **g**) injection (20 μL) and monitored at 2 (**a**, **c** and **g**), 24 (**b**, **d** and **g**) and 48 h* (**e**, **f** and **g**) after therapy. Original magnification × 1000 for all panels. Bars represent 20 μm

**Fig. 9:**
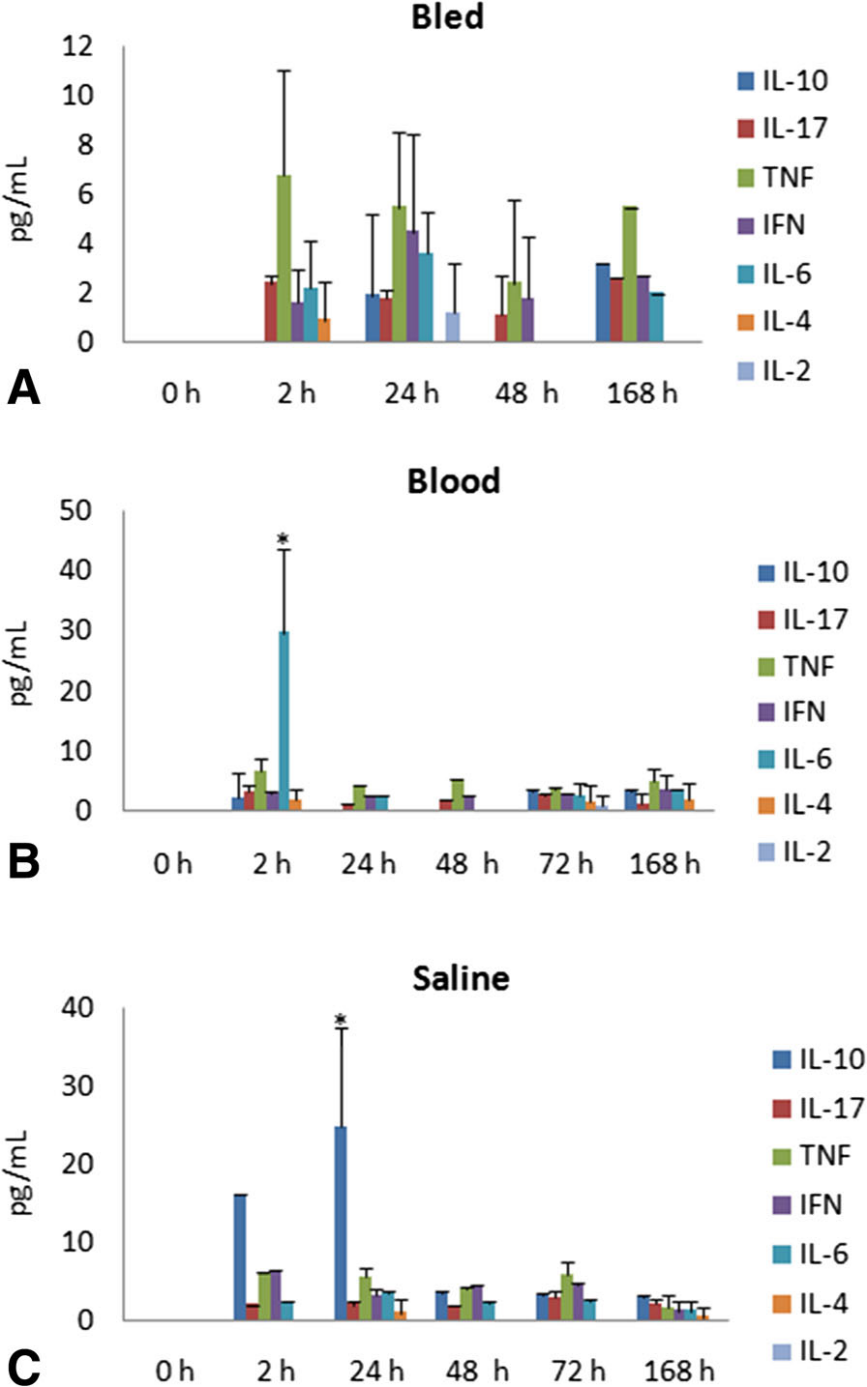
Analysis of the plasma cytokine profile in uninfected mice. Mean and SD of cytokine blood samples from mice that were only bled (**a**) or submitted to AWB (**b**) and SAL (**c**) injection. The samples were collected at endpoints of 2, 24, 48, 72 and 168 h after treatment (single administration - Scheme 2). * ANOVA = *p* ≤ 0.05 (*n* = 2–3) between bleeding/treated animals

While investigating the inflammatory profile we found a difference in inflammatory response kinetics when SAL and AWB administration was performed. After 2 h, a stronger inflammatory response was observed at the sites of muscle inoculation with AWB (Figs. 4b, 5b, 6b and 7b) as compared to SAL exposition (Figs. 4b, 5b, 6b and 7c), with this difference being maintained until 24 h (Figs. 4c, 5c, 6b and 7). When the applied volumes were fractionated (10 + 10 and 20 μL), differences were found in both parameters when both blood and saline were given especially starting at 24 h after injection: number of infiltrates and lesion grade (Figs. 4 and 5). Furthermore, up to 24 h, all studied groups (except one mouse from the 20 μL AWB group) showed a predominance of polymorphonuclear cells (data not shown). After 48 h, there was an inversion of this inflammatory profile, being (in all groups) predominantly mononuclear (data not shown). SAL groups revealed higher levels of tissue inflammation from 48 h up to the last day studied (168 h) while at this latest time-point, the lesion intensity and number of inflammatory cells decreased in the AWB groups (Figs. 4, 5 and 7). Aiming to identify the presence of eosinophils, Sirius red labeling was performed. This dye has high affinity for existing cytoplasmic granules in eosinophils. Thus, it was possible to carry out their quantifications individually. The histopathological analysis (AWB and SAL 20 μL) using Sirius red showed that after a single administration, an eosinophil migration occurred after 2 h, being at 24 h higher in the blood-injected mice as compared to SAL intervention (21.5 and 7%, respectively) (Fig. 8). Our findings also showed in one out of five mice that received three times 20 μL of AWB presented a higher level (2.7-fold) of polymorphonuclear cells as compared to one out of three mice that received only one AWB administration (Fig. 8). Analysis of cytokines by flow cytometry performed at 2, 24, 48, 72 and 168 h after administration showed respective peaks of IL-6 and IL-10 after 2 and 24 h when AWB (Fig. 9b) and SAL (Fig. 9c) were evaluated (Fig. 9).

### Analysis of AWB administration in mice experimentally infected by *T. cruzi*

**Fig. 10:**
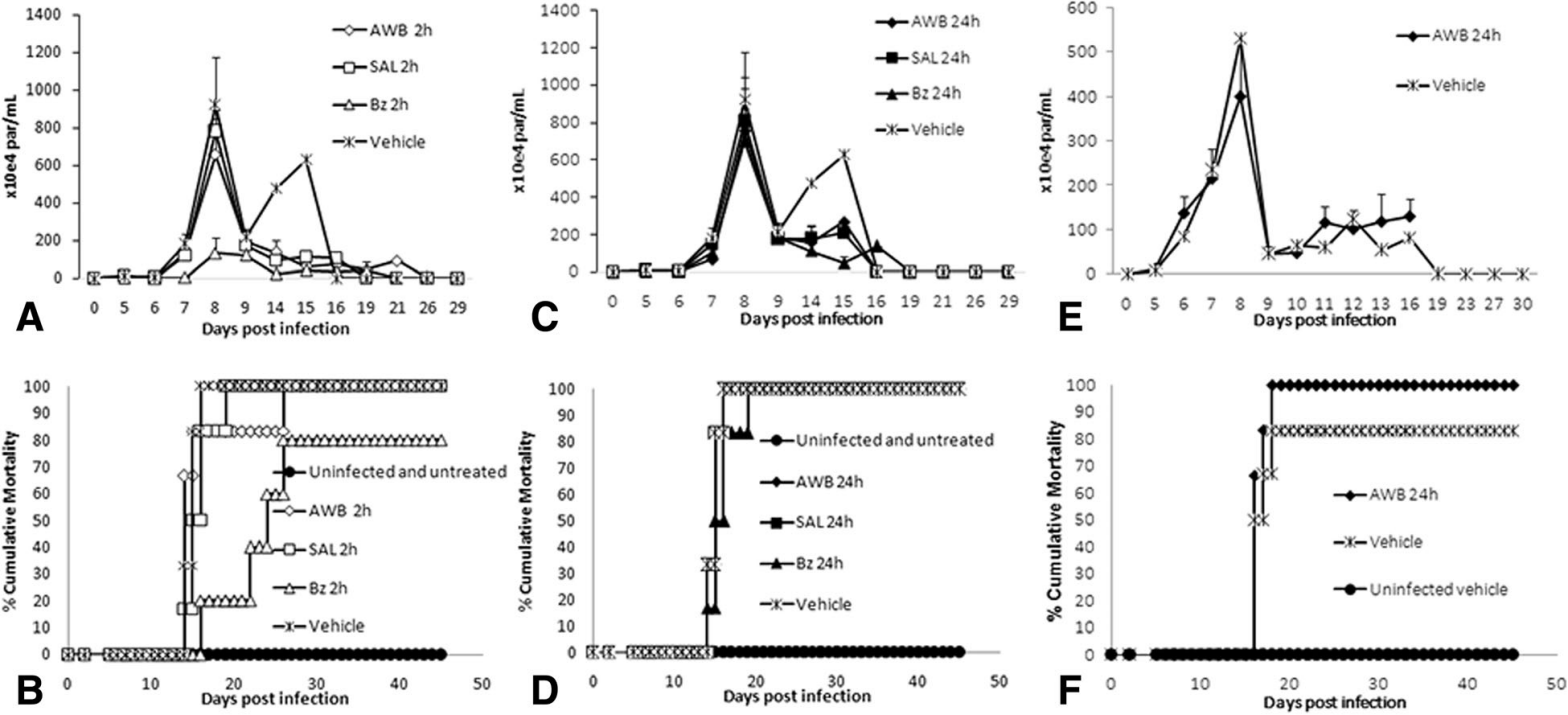
Analysis of parasitemia levels and percentage of cumulative mortality in acute *T. cruzi* infection of mice. *In vivo* effect of single (**a**–**d**) and three (intervals of 5 days between each dose) (**e**, **f**) administrations of autologous whole blood (AWB), saline (SAL) and benznidazole (Bz) prior to *T. cruzi* acute infection using male Swiss mice inoculated with 10^4^ bloodstream trypomastigotes (Y strain). The data express parasitemia levels (**a**, **c** and **e**) and percent of cumulative mortality (**b**, **d** and **f**)

Our first approach was the standardization of AWB protocols to be used before (prophylactic) and after (therapeutic) parasite infection. The findings from one AWB and Bz administration provided before the parasite inoculation demonstrated that only the reference drug given at 2 h prior to infection was able to significantly (*p =* 0.02) reduce (86%) the parasitemia peak (at 8 dpi, in this experimental model) (Fig. 10a). AWB groups only presented minor alteration in the parasitemia levels, leading to decreases of 29% and 18% at 2 h and 24 h prior to infection, respectively, reaching similar levels as Bz administered before 24 h (Fig. 10a and c). Despite this, only the Bz-treated group at 2 h prior to infection was able to confer animal survival of 20% while all other mouse groups reached 100% death similarly as did the vehicle-treated mice (Fig. 10b and d). Since a mild reduction was produced by only one injection of AWB, our next step was to check whether multiple administrations could improve the anti-parasitic effect. In this sense, consecutive AWB administrations (three injections at five-day intervals), with the last dose being just 24 h before parasite infection. Our data showed that three administrations of AWB produced results similar to a single blood injection, reaching a maximum decrease of 24% at the parasitemia peak (*p* ≤ 0.05) (Fig. 10e), failing to protect against death triggered by the parasite infection (Fig. 10f).

**Fig. 11:**
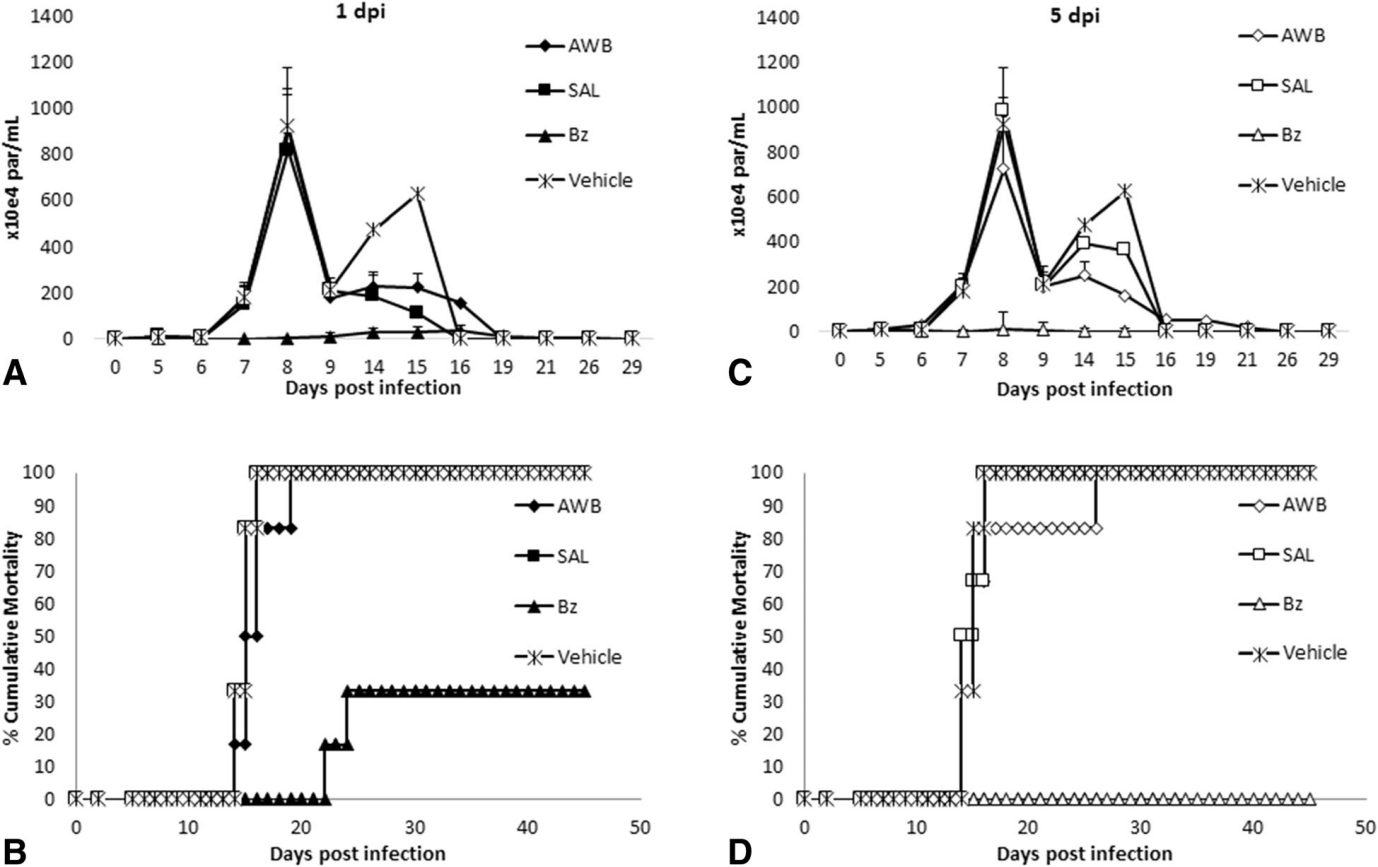
Analysis of parasitemia levels and percentage of cumulative mortality in acute *T. cruzi* infection of mice. *In vivo* effect of autologous whole blood (AWB), saline (SAL) and benznidazole (Bz) administration for 5 consecutive days after *T. cruzi* acute infection using male Swiss mice inoculated with 10^4^ bloodstream trypomastigotes (Y strain). **a** and **c** parasitemia levels and **b** and **d** percent of cumulative mortality. The therapy was started at 1 dpi (**a** and **b**) and at the parasitemia onset (5 dpi – **c** and **d**). dpi = Days post infection

**Fig. 12:**
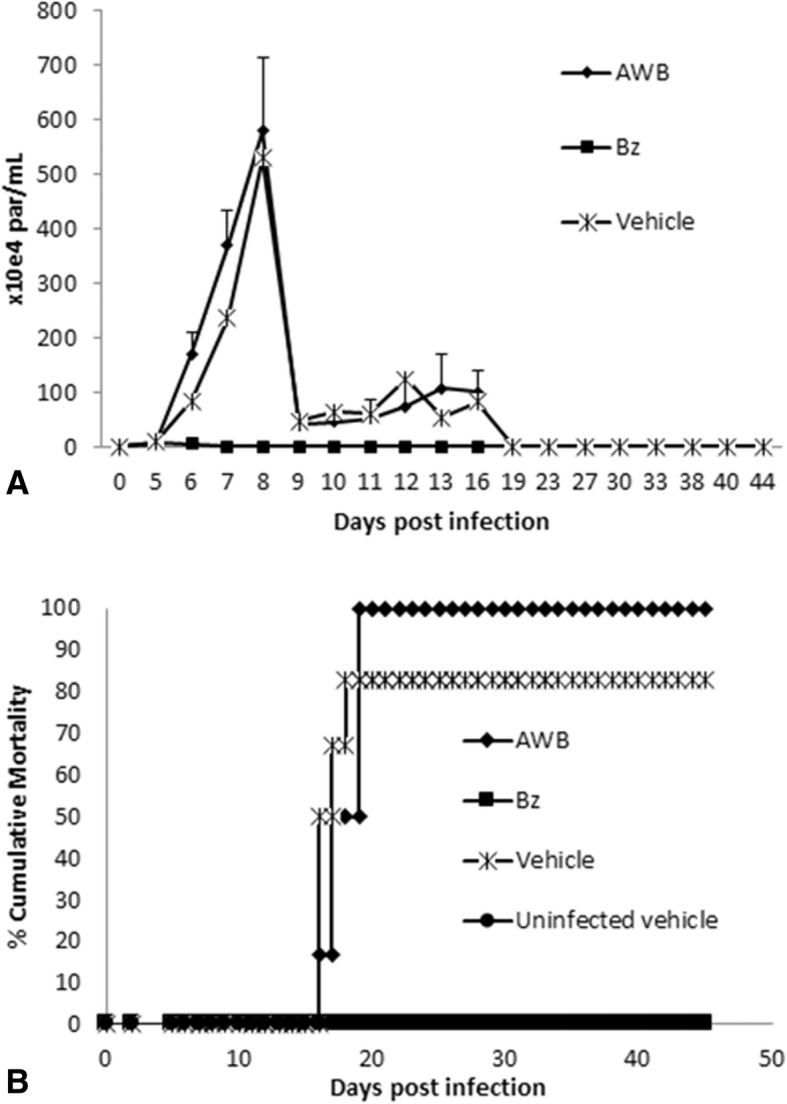
Analysis of parasitemia levels and percentage of cumulative mortality in acute *T. cruzi* infection of mice. *In vivo* effect of autologous (AWB) and benznidazole (Bz) administration for 10 consecutive days after *T. cruzi* acute infection using male Swiss mice inoculated with 10^4^ bloodstream trypomastigotes (Y strain), evaluated through parasitemia levels (**a**) and percent of cumulative mortality (**b**). The therapy was started at the parasitemia onset (5 dpi)

Concomitant to the evaluation of potential AWB as prophylactic anti-parasitic approach through its use prior to parasite inoculation, we also studied the potential effect of this practice *in vivo* after infection. In this analysis, multiple consecutive AWB administrations were given to infected animals starting the therapy using a preventive (at 1 dpi) or therapeutic (at 5 dpi that represents the parasitemia onset in this experimental model) protocol [41]. In both cases, no significant effect on the parasitemia levels was observed (Fig. 11a and c) and all animals died (Fig. 11b and d). Bz treatment was able to suppress infection completely (Fig. 11a and c) besides protecting against mortality (Fig. 11b and d). To further evaluate whether longer AWB administration could improve its potential effect upon *T. cruzi* experimental infection, another set of assays were conducted extending the use of AWB for 10 consecutive days. The results showed that only the reference therapy performed with Bz was able to suppress the parasitemia and also provide 100% survival of mice (Fig. 12a and b).

**Fig. 13:**
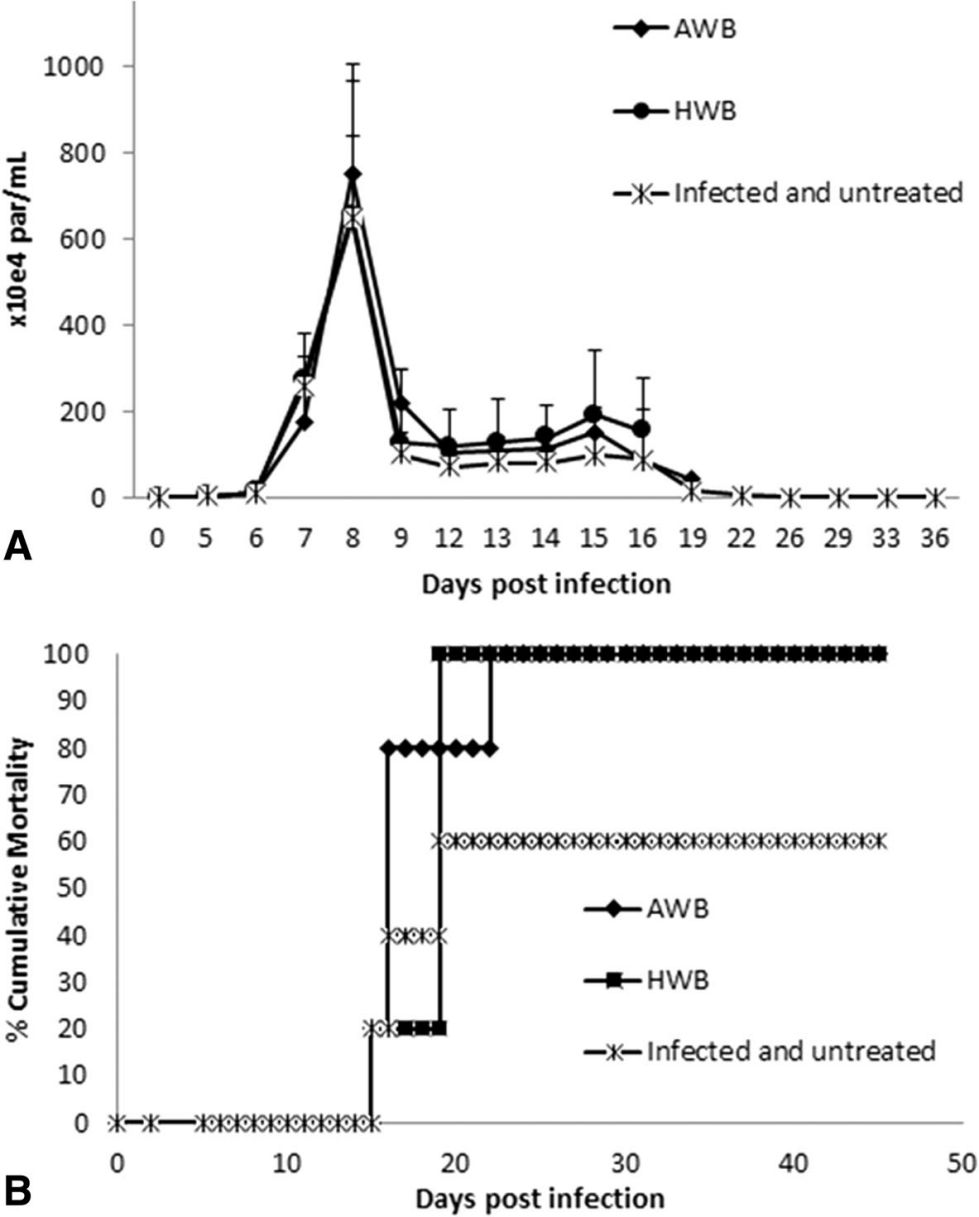
Analysis of parasitemia levels and percentage of cumulative mortality in acute *T. cruzi* infection of mice. *In vivo* effect of three (intervals of 5 days) administration of AWB, HWB and benznidazole prior *T. cruzi* acute infection using male Swiss mice inoculated with 10^4^ bloodstream trypomastigotes (Y strain) evaluated through parasitemia levels (**a**) and percent of cumulative mortality (**b**)

**Fig. 14:**
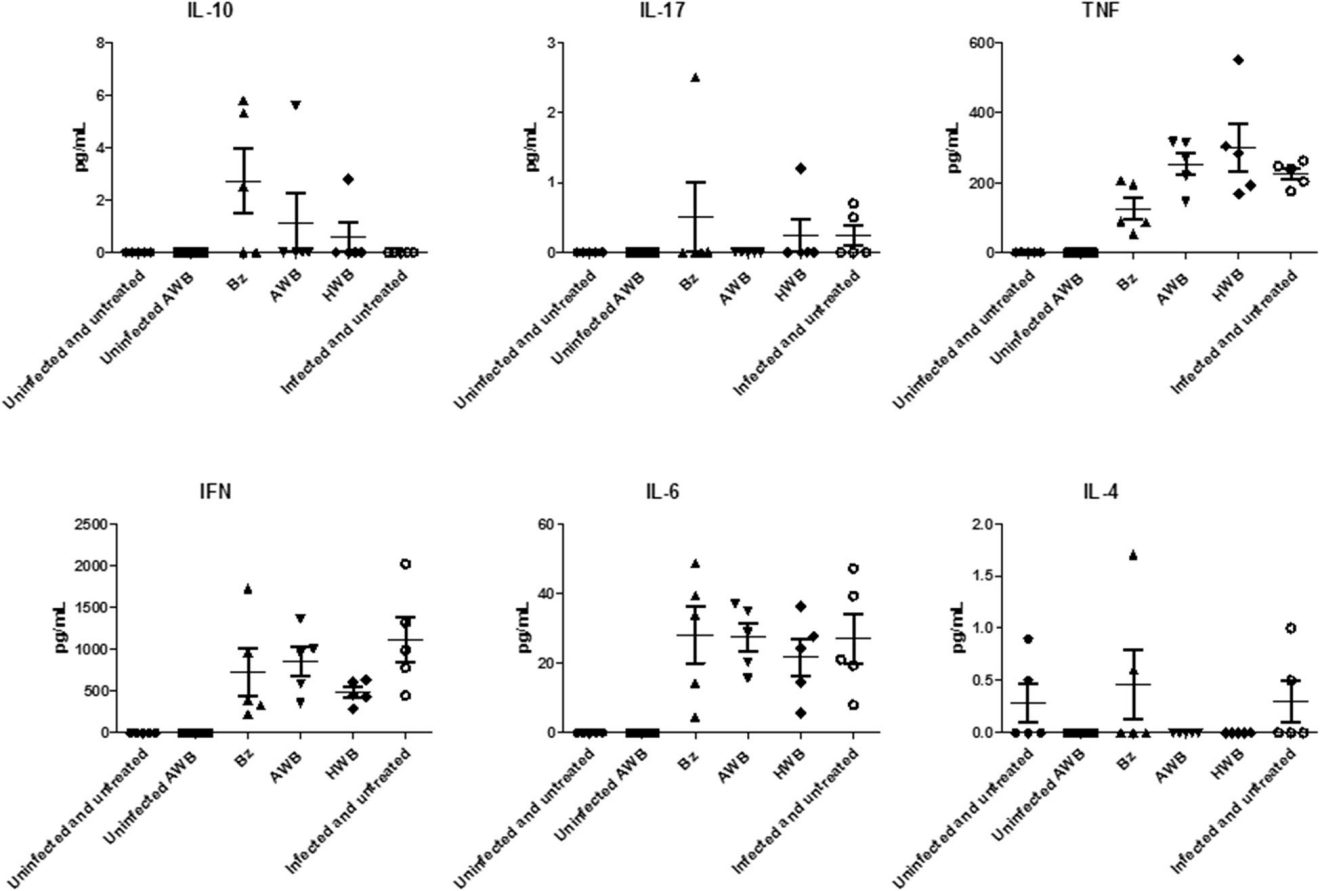
Analysis of the plasma cytokine profile in acute *T. cruzi* infection of mice. The blood samples were collected at 9 dpi from mice submitted to three AWB or HWB (intervals of five days) administrations prior to *T. cruzi* acute infection or benznidazole (5 and 9 dpi) using male Swiss mice inoculated with 10^4^ bloodstream trypomastigotes (Y strain)

**Fig. 15:**
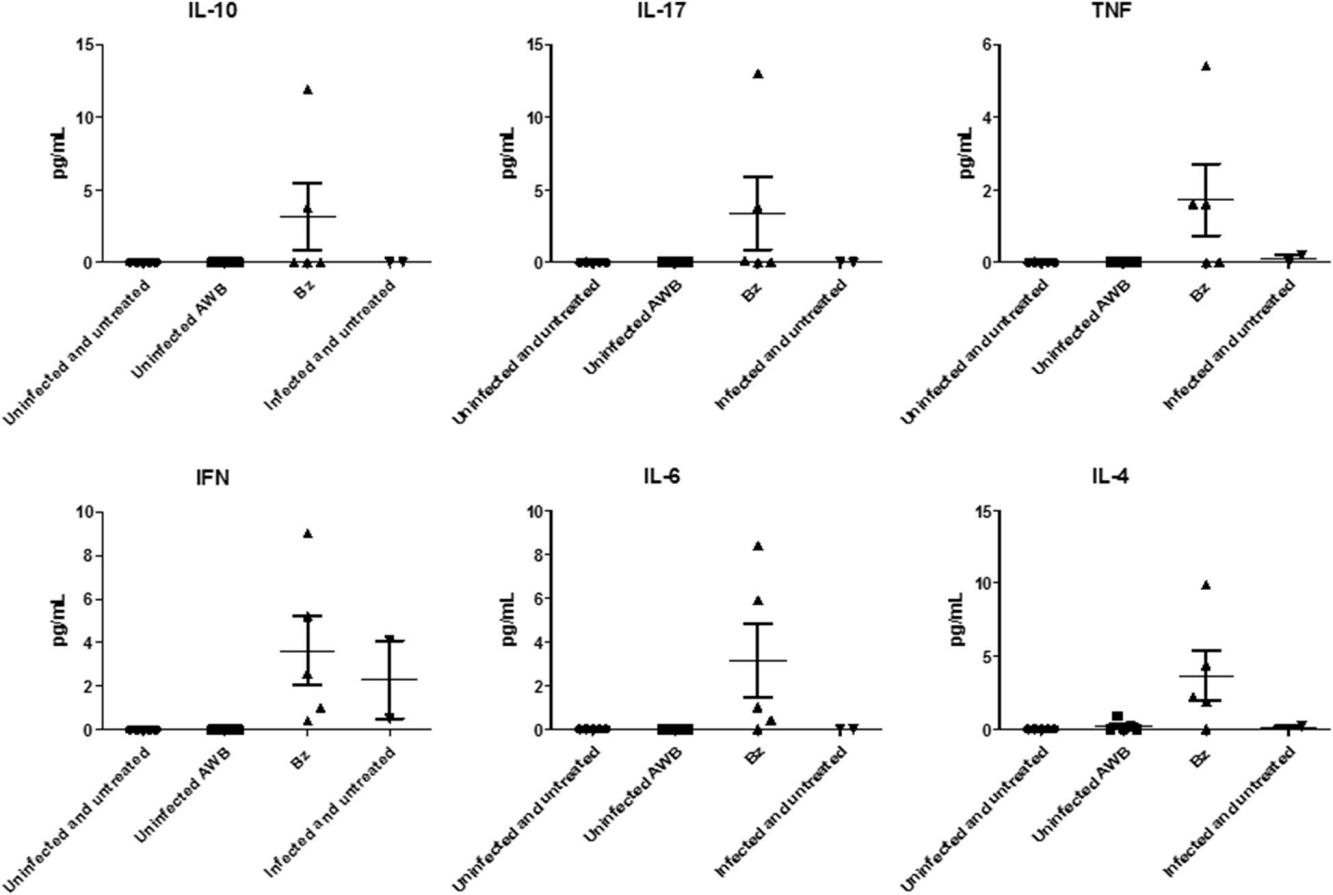
Analysis of the plasma cytokine profile in acute *T. cruzi* infection of mice. The blood samples were collected at 40 dpi from mice submitted to three AWB or HWB (intervals of five days) administrations prior to acute *T. cruzi* infection or benznidazole (5 and 9 dpi) using male Swiss mice inoculated with 10^4^ bloodstream trypomastigotes (Y strain). *ANOVA = *p* ≤ 0.05 (*n* = 5) related to infected and untreated. Dpi = Days post infection

**Fig. 16:**
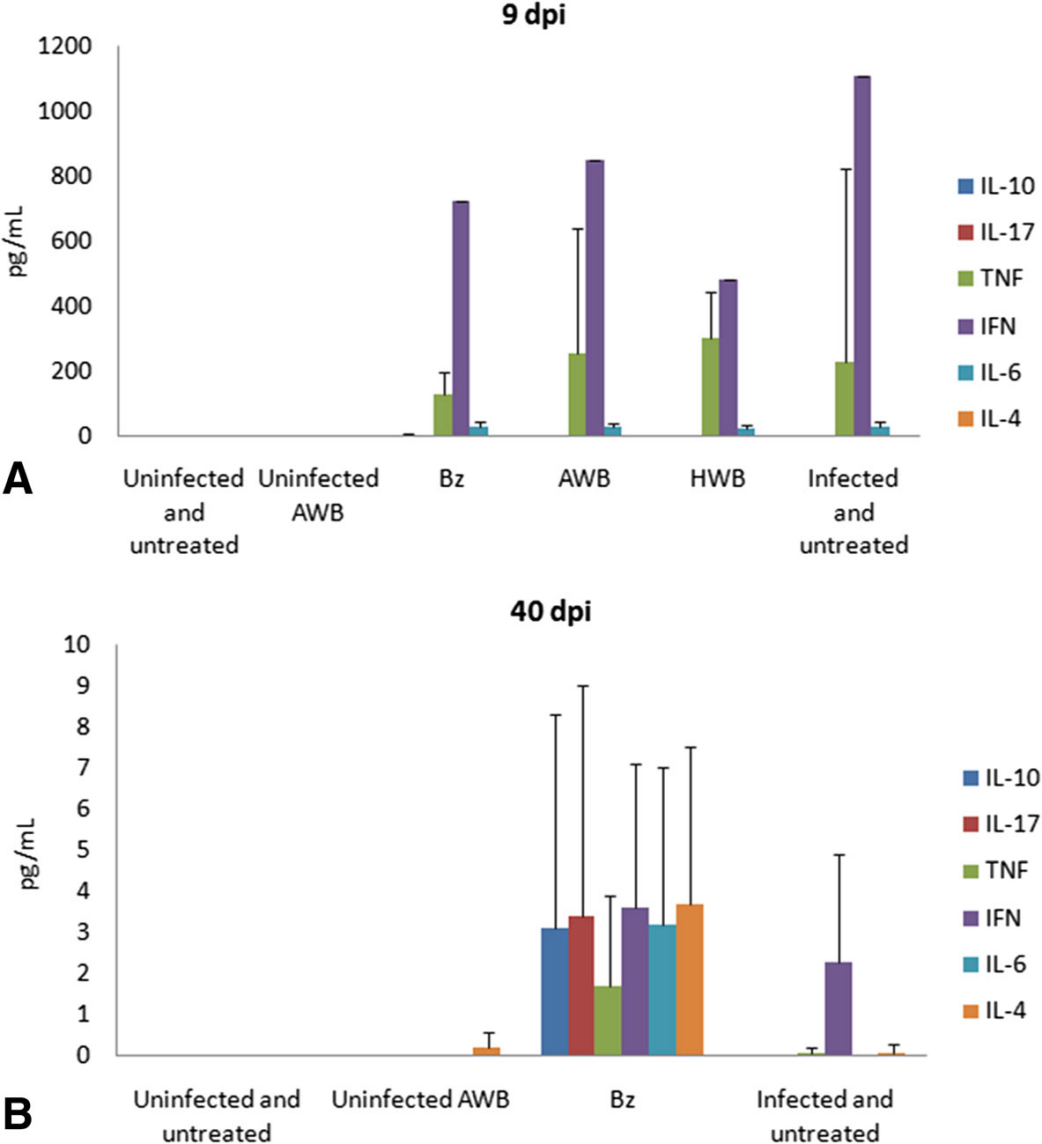
Analysis of the plasmatic cytokine profile at 9 (**a**) and 40 (**b**) days post infection of mouse models inoculated with bloodstream forms of Trypanosoma cruzi (Y strain). Mean and SD of the cytokines from the animal blood samples are given after three AWB or HWB administrations prior to parasite inoculation. Benznidazole was also given (from 5 to 9 dpi) as reference drug

Finally, as we found increased levels of inflammatory mediators due to AWB doses administered in healthy animals (Fig. 9), the analysis of the plasma profile was conducted using infected mice exposed or not to AWB as well as heterologous whole blood (HWB). Uninfected-and-untreated, infected-and-untreated, uninfected-and-AWB-treated and Bz-treated mouse groups were evaluated as control groups (Figs. 13, 14, 15, and 16). Inflammatory profile analysis was carried out at 9 dpi and at 40 dpi (surviving animals) (Figs. 14, 15 and 16). The findings showed that major alterations occurred only at the acute parasitemia peak period (corresponding to 9 dpi) due to increased levels of IFN-gamma (5500-fold), TNF-alpha (754-fold) and IL-6 (260,000-fold) in untreated and infected mice as compared to uninfected and untreated animals (Fig. 16). Regarding the treated groups, at the 9 dpi, only in Bz-treated animals, TNF-alpha levels were significantly (*p =* 0.02) decreased as compared to infected and untreated mice group (Fig. 16). The presence of IL-2 was not detected in any of the studied groups. As to the ponderal curve, except for those animals treated with Bz post-infection, all infected animals displayed weight loss in the second week of infection (*p* ≤ 0.05) (data not shown). As found for AWB, the therapy using HWB did not reduce the parasitemia levels neither protect against mice mortality (Fig. 13a, b), while Bz (given at 5 and 9 dpi) completely diminished the parasitemia and impaired mice mortality.

## Discussion

Although the use of AWB has been described as treating allergic rhinitis, autoimmune pathologies, osteoarthritis, atopic dermatitis, and infectious diseases, it still represents a controversial issue due to the very limited pre-clinical and clinical studies besides the lack of knowledge regarding its action mechanism [21, 27, 48]. In this sense, we aimed to investigate clinical, biochemical and histopathological aspects of the AWB intervention (im) using healthy mice and *T.cruzi*-infected mouse models under different schemes such as: (i) single and multiple (three) administrations, (ii) different volumes of blood fractioned (10 + 10 μL) or not, comparing to the findings found for control groups (neither bled nor injected and bled but not injected, mice inoculated with saline). The maximum volume (20 μL) was based on previous assays in other animal models including rats (v/g animal mass weight not exceeding 1 mL/kg) [40], taking also into consideration a similar proportion (*v*/v) described in the folk literature and a few clinical trials [23, 25]. Our findings related to behavioral parameters showed that although animals treated with one or three injections of AWB or saline showed no significant changes, in 20% (4 out of 20 mice) of those that received 20 μL for three times presented an altered paw gait. Our data corroborate results found in another experimental model (rat) of repeated autologous intraarticular blood injection that provoked a pain-related behavior [49]. For a period of 50 days, animals were injected weekly in one knee joint with either whole blood or cellular/plasma components; the results demonstrated primary hyperalgesia starting after the third injection of whole blood samples, accompanied by mild functional gait changes [49]. The authors found that this side effect was most prominent in whole blood injected animals as compared to plasma injection and thus the effect of the cells may be additive in promoting pain. Furthermore, they reported that animals, which received whole blood only once, did not present any gait alterations [49], corroborating our present data. In clinical trials, the most frequent patient complaint after the intraarticular injection of platelet-rich plasma (PRP) was pain at the injection site that in some cases lasted up to 10 min after injection, decreasing gradually but in others continued up to 2 weeks [50]. Except for the gait impairment, no other major side effect was noted including evidence of altered biochemical analysis or modifications in the size or gross pathology of the studied organs (heart, liver, spleen and kidneys) after AWB injection. In addition, in order to reduce the possible impact of the needle size used in AWB inoculation (although in the SAL group we did not observe gait impairment after a similar administration volume), a smaller caliber needle was used in all subsequent studies.

In healthy animals, a huge inflammatory cell infiltration was induced by AWB at the injection site at 2 h after injection, being stronger and earlier as compared to saline. In parallel, we detected a rapid increase in IL-6 levels, a pro-inflammatory mediator that acts as systemic activator of acute phase proteins [51]. On the other hand, after 24 h animals treated with SAL presented an increase in IL-10 levels, a mediator essential in the maintenance of tissue integrity during the inflammation process caused by infections or lesions [52]. A different hypothesis might be proposed to explain the huge inflammatory response localized at the injection site. This response may be triggered by the exposure to self-antigens that were inoculated at muscle sites in areas where constituents of blood antigens should not be presented under physiological conditions. Furthermore, the mechanical injury induced by the needle introduction or liquid volume administration leading to tissue disorganization may also, at least in part, include the migration of inflammatory cells, as was found when saline was given as a vehicle [53].

Inflammation is controlled by several extracellular mediators, including cytokines, growth factors, eicosanoids, complement and peptides among other molecules. Cytokines are key modulators of inflammation, participating in acute and chronic phases [54, 55]. These proteins have a specific effect on the interactions and communications between cells and mediate a wide variety of biological activities, such as inflammation induced by an immune response, as well as tissue repair and remodeling [54]. Pro-inflammatory cytokines predominantly are produced by activated macrophages and are involved in the up-regulation of inflammatory reactions [54]. This cytokine class includes IL-1, IL-6 and TNF-alpha. TNF alpha is an important mediator for the inflammatory action of the innate immune system, participating in the induction of cytokine production, activation of adhesion molecules, and growth stimulation. IL-6 is involved in hematopoiesis, and is critical in the antibody production by B cells, activation of T cells, differentiation and regulation of Th2 and Treg phenotypes. It also plays a role in the secretion of acute-phase proteins [51, 55]. In turn, different mechanisms provide the fine-tuning of inflammation and a favorable environment for the resolution to take place and for homeostasis to return. Resolution of inflammation is orchestrated by a large panel of mediators that act by controlling the pro-inflammatory cytokine response [54, 56]. IL-10 is a potent anti-inflammatory mediator that represses the expression of inflammatory cytokines by a different population of activated macrophages [52, 54].

One interesting histopathological finding in the present work was the rapid tissue in situ repair after a week of administration interruption. Although a strong inflammatory profile was noted in AWB-treated mice, a higher level of eosinophils (stained by Sirius red) was found in one mouse after three injections as compared to another animal that received a single inoculum. This difference also needs to be explored in greater depth to evaluate the potential role of these cells in the inflammatory context triggered by AWB interventions. A recent study reported that single or double injections of PRP in patients suffering from knee arthritis resulted in similar clinical benefits in both protocols exhibiting better effects than injection of normal saline [50].

With respect to CBC analysis in healthy animals, 48 h after the third administration of AWB, the WBC and RBC values were lower in all groups submitted to any type of bleeding intervention as compared to those not bled and the untreated control. According to Hoff [57], in mouse models, although the blood volume can be replaced within 24 h of bleeding, the number of erythrocytes is only completely restored in up to two weeks. As higher amounts of growth factors and cytokines are present in platelets while the plasma displays proteins and bioactive molecules (playing roles in the cellular repair process), randomized clinical trials using intraarticular injection of PRP to treat chronic progressive pathologies such as osteoarthritis have been done based on the hypothesis that it could regulate anti-inflammatory signals and equilibrate angiogenesis [50]. Presently, only AWB samples collected without anticoagulants were investigated, which precludes us from disclosing the role of the platelet itself in the inflammatory state. Therefore, future analysis needs to be conducted to verify the impact of PRP in our mouse model compared to the AWB schemes (with or depleted of platelets). The role of not only platelets but also monocytes and white cell stem cells in AWB and PRP has been widely discussed; it has been proposed that leukocyte secretion of proteases and reactive oxygen may be undesirable for the therapy of chronic pathological conditions. Other authors have asserted that the secretion of substances such as cytokines and enzymes may be effective in the processes of repair, platelet activation, prolonging the duration of growth factor release and prevention of infection [58]. Comparing the levels of cellular components in healthy mice, we observed that platelet levels were lower when three interventions were performed, which may suggest a higher recruitment towards the sites of injury/inflammation when there is higher tissue damage (e.g. three AWB injections). Platelets are capable of not only interacting with the leukocytes and endothelial cells, but also promoting the formation of a blood clot [58]. As demonstrated in previous studies using the same relation of v/g in a rat model, the volume presently applied did not affect blood oxygenation given that neither cyanosis nor reduced blood hemoglobin levels were found [40]. Interestingly, an intense inflammatory infiltrate was present in all healthy animal groups, except for those that did not receive any type of intervention or were only bled. In the administration of blood and saline, a predominant polymorphonuclear infiltration was observed until the time of 24 h, and was subsequently replaced by mononuclear cells. The high concentration of macrophages at the site of the muscle injury may act on the regulation of satellite cell mitotic activity, giving rise to new muscle fibers, and in addition, leading to the release of growth factors [59]. The degree of lesions and inflammation was related to the applied volume of SAL and AWB being higher in the groups that received 20 μL, thus also corroborating the idea that the lesion degree can be related to the volume of solution administered intramuscularly.

In the field of pathologies caused by infectious agents, few data are available. Some studies suggest that AWB may induce protection against viral infections, thus contributing to a rapid improvement of clinical status in patients [26] and animals [24, 25].

In this context, we aimed to investigate the potential effect of AWB administration in the course of a parasitic pathology using a mouse model of acute *Trypanosoma cruzi* infection, the intracellular obligate parasite that causes Chagas disease (CD), also known as American trypanosomiasis. CD is endemic in 21 Latin America countries, where it represents a significant ischemic and inflammatory heart disease [60]. About 6 million people are affected worldwide, with approximately 10 thousand annual deaths and more than 25 million individuals under risk of infection [17]. The disease also occurs in such non-endemic areas as Europe, Asia and North America, mainly due to the migration of infected individuals [17, 39]. Nifurtimox (Nf) and Benznidazole (Bz) are the only drugs available to treat the disease and were developed more than five decades ago. Besides their significant toxicity, which leads to discontinuation of treatment for many patients, both are effective only for the acute phase of the infection. Because of this, the development of new therapeutic approaches is urgently needed [39, 61].

In order to verify whether intramuscular AWB procedure can impact the course of a parasitic disease, different assays were presently explored under prophylactic (prior the infection) and therapeutic (post infection) schemes conducted under distinct periods of AWB administration (from one up to 10 days). The findings were also compared with those obtained using heterologous (HWB) blood. The sum of our results demonstrated that in all studied schemes, only minor decreases (< 30%) in the parasitemia levels were found when AWB was given. It is possible that this mild decrease in the parasitemia levels (18–29%) may be related to temporary and early increases of IL-6 levels as we observed in healthy AWB-treated mice. According to the properties of this cytokine described above, it might play a role in partially reducing the parasite burden in AWB exposed-animals. On the other hand, our positive therapeutic control performed with the reference drug (Bz) was able, as expected by the use of its optimal dose [41] to suppress the blood parasite load and protect against the mortality rates induced by this parasitic infection in this experimental model. AWB and HWB (single and multiple doses) given before and after parasite infection did not increase animal survival, and presented similar mortality levels as both untreated and vehicle-treated infected animals.

In order to investigate whether AWB or HWB would be able to trigger an inflammatory response different from that normally presented in an acute infection model and, in addition, to conduct a second analysis of the cytokine profile in healthy animals using a different treatment protocol, the cytokine panel was evaluated. We found rises in IFN-gama, TNF-alpha and IL-6 at 9 day after infection in all infected groups as compared to uninfected mice but only Bz displayed statistically significant lower (*p =* 0.02) TNF-alpha levels, possibly due to reduced parasitism levels and respective antigenic stimuli. Our cytokine findings corroborate previous studies using murine models of acute infection that reported elevated levels of proinflammatory cytokines IFN-gamma, TNF-alpha, and IL-6 in untreated and infected animals compared to uninfected mice and that Bz reduced the plasmatic levels of these cytokines [62, 63]. As to the ponderal curve, only animals treated with Bz after infection showed protection against weight loss, while the other presented a decrease in weight gain. Our data also confirmed a previous analysis using the same experimental model in which Bz therapy restores the animal weight as compared to infected and untreated mice [64].

The literature reports on the potential benefit of AWB towards microorganism infections are very scarce and display controversial results. Mettenleiter [65] suggested that the use of AWB (single intervention) may act as a prophylactic treatment, especially in the prevention of postoperative pulmonary complications in patients submitted to different surgical procedures. Parvovirus-infected dogs that were submitted to AWB treatment showed signs of more effective recovery, besides a lack of side effects [21]. On the other hand, using the same methodology, Ottobelli et al. [38] showed that this procedure has no influence on the leukocyte levels. In addition, platelet-rich plasma (PRP) has been recognized as a support procedure due to the presence of growth factors and other biomolecules promoting endogenous microbicidal activity. However, a recent study using PRP on the sutured skin of randomized patients undergoing foot or ankle surgery demonstrated that this procedure was unable to reduce the incidence of postoperative infection [66].

The results obtained in the present study revealed that the use of autologous whole blood in acute model of *T. cruzi* infection under the experimental conditions presently performed was unable to reduce the parasitic load of infected mice, providing only a minor decrease in parasitemia levels (up to 30%) but without protecting against animal mortality. In this sense, it is important to investigate this practice in greater depth to elucidate the potential role and use of AWB for future clinical therapeutic purposes.

## Conclusions

Our data encourage additional experimental research regarding the administration of autologous blood in order to further explore its potential protective effect during pathological states such as those induced by an infectious agent. In this sense, the potential impact on the inflammatory response in the course of such a pathological state (e.g., one caused by parasitic infections) merits additional investigation given that alternative therapies may be added to the etiological discovery process to improve the life quality of the patients.

## Abbreviations

ACMPs: Alternative or complementary medical practices
ALT: Alanine aminotransferase
AST: Aspartate aminotransferase
AWB: Autologous whole blood
BT: Bloodstream tripomastigote
BUN: Urea
Bz: Benznidazole
CBC: Complete blood count
CK: Creatine kinase
dpi: Days post infection
HE: Hematoxylin-eosin
HWB: Heterologous whole blood
ia: Intra-arterial
iar: Intraarticular
IFN: Interferon
IL: Interleukin
im: Intramuscular
iv: Intravenous
Nf: Nifurtimox
PRP: Platelet-rich plasma
pt.: Post treatment
RBC: Red blood cell
SAL: Saline
sc: Subcutaneous
TNF: Tumor necrosis factor
WBC: White blood cell
